# Supplementary material for: Multistage lithospheric drips control active basin formation within an uplifting orogenic plateau
Source: Nat Commun. 2024 Sep 13;15:7899. doi: 10.1038/s41467-024-52126-7 (PMC11399430; doi:10.1038/s41467-024-52126-7)
Supplement: Supplementary file 1 — Supplementary Information [file 41467_2024_52126_MOESM1_ESM.pdf]

Multistage lithospheric drips control active basin formation within an uplifting orogenic plateau

Supplementary Information

Additional data show residual topography profiles and crustal thickness variations along a N-S profile through the Konya Basin region (Supplementary Figure 1).

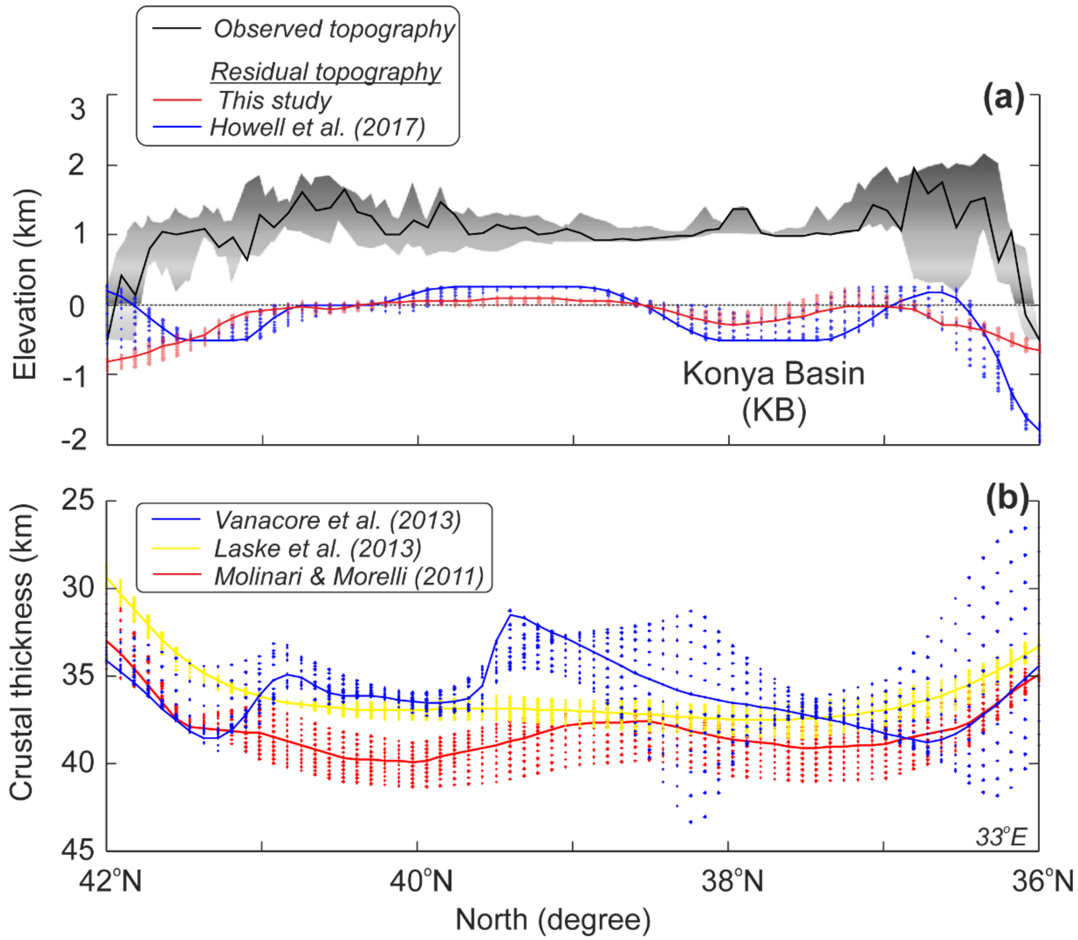

Supplementary Figure 1 | Cross-sections of residual topography and crustal thickness data **a)** Residual topography from (Howell et al., 2017)[1] in comparison with our residual topography calculation (see main text). **b)** Crustal thickness variations along a 1° swath profile centered 33°E. Minimum and maximum values are shown in grayscale color and dotted shaded area (solid lines show the data along 33°E [2–4]).

3-D analogue modeling studies by Pysklywec & Cruden (2004) suggest [5] that lithospheric drips may be categorized as either symptomatic or asymptomatic based on their surface tectonic responses. Symptomatic drips show a higher degree of coupling between the descending mantle lithosphere and the overlying crust. For example, models show that the crust can be pulled inwards by the sinking lithosphere while producing crustal shortening including wrinkle ridges and fold-and-thrust belts (Supplementary Figure 2a). On the other hand, asymptomatic drips are not well coupled to the uppermost mantle lithosphere and overlying crust and yield little to no accumulation of crustal deformation (Supplementary Figure 2b). However, these asymptomatic local downwellings may induce crustal subsidence or uplift [6].

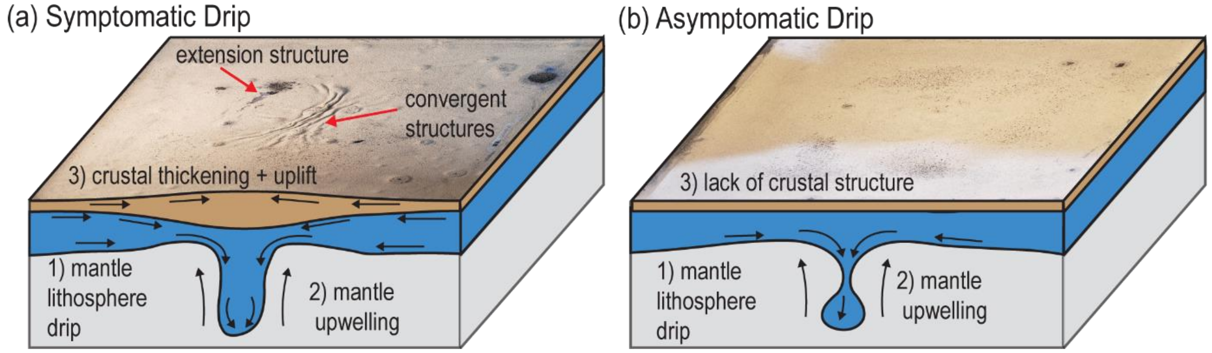

Supplementary Figure 2 | Symptomatic vs. asymptomatic drip concept illustration a) Illustration of a symptomatic drip with thickening and uplift in the upper crust from horizontal convergence and extension. Modified from (Andersen et al., 2022), Fig. 4 [6] and (Pysklywec & Cruden, 2004), Fig. 1 [5]. **b)** Illustration of an asymptomatic drip where the mantle lithopshere dripped beneath the upper crust but there are no convergent structures in the crust that indicate such a process occurred. Modifed from (Pysklywec & Cruden, 2004) Fig 1 [5].

Supplementary Figure 3 shows the surface elevation contours during the earlier phase of the experiment while the primary drip is descending at 10 and 25 hours (Supplementary Figure 3a, b). The surface at 50.6 and 57.1 hours (corresponding to Figure 3e,g but plotted at a new scale) are

also included and these show the resurgence of subsidence associated with the development of the secondary drip pulse. The surface subsided rapidly at earlier stages before the camera was turned on creating a basin, which became shallower over time as the primary drip thinned and progressed towards the bottom of the tank. The results show that the geometry and location of the secondary drip topography is not the same as with the primary drip: notably rather the centre of the secondary drip depression has shifted downwards in this plan frame.

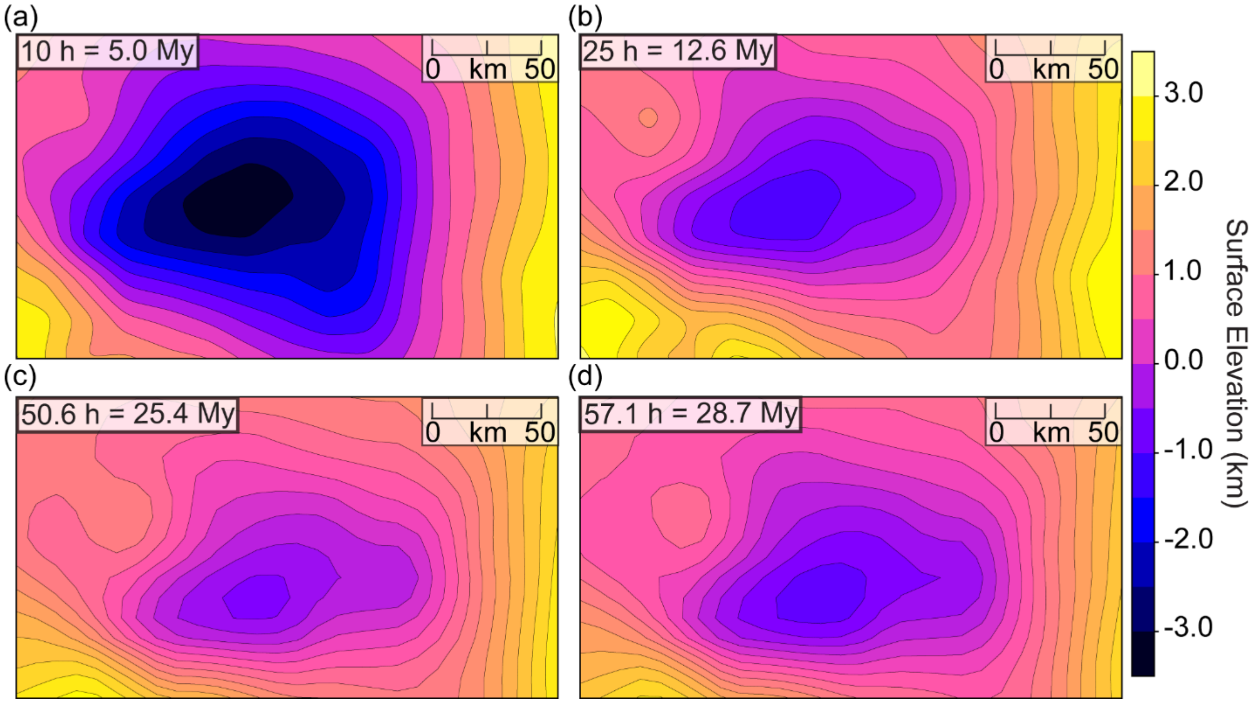

Supplementary Figure 3 | Topographic evolution of EXP-1 from 10 h to 57.1 h a) Surface elevation of EXP-1 at 10 h following the development of the primary drip. A deep (~3.0 km) basin has developed at this stage. **b)** Surface elevation at 25 h. The basin created by the primary drip has uplifted as the drip thinned. **c)** The basin formed by the primary drip continued to uplift to its shallowest point at 50.6 h. At this point the secondary drip has begun to descend. **d)** At 57.1, the secondary drip has pulled the surface downward, deepening the basin again.

References

1. Howell, A., Jackson, J., Copley, A., McKenzie, D. & Nissen, E. Subduction and vertical coastal motions in the eastern Mediterranean. *Geophys J Int* **211**, 593–620 (2017).
2. Vanacore, E. A., Taymaz, T. & Saygin, E. Moho structure of the anatolian plate from receiver function analysis. *Geophys J Int* **193**, 329–337 (2013).
3. Laske, G., Masters, G., Ma, Z. & Pasyanos, M. Update on CRUST1. 0—A 1-degree global model of Earth's crust. in *EGU General Assembly* 2658 (Geophysical Research Abstracts, Vienna, Austria, 2013).
4. Molinari, I. & Morelli, A. EPcrust: A reference crustal model for the European Plate. *Geophys J Int* **185**, 352–364 (2011).
5. Pysklywec, R. N. & Cruden, A. R. Coupled crust-mantle dynamics and intraplate tectonics: Two-dimensional numerical and three-dimensional analogue modeling. *Geochemistry, Geophysics, Geosystems* **5**, Q10003 (2004).
6. Andersen, J., Gögus, O. H., Pysklywec, R. N., Santimano, T. & Uluocak, E. Ş. Symptomatic lithospheric drips triggering fast topographic rise and crustal deformation in the Central Andes. *Commun Earth Environ* **3**, 1–12 (2022).
